# Palm Oil (*Elaeis guineensis*): A Journey through Sustainability, Processing, and Utilization

**DOI:** 10.3390/foods13172814

**Published:** 2024-09-04

**Authors:** Adamu Muhammad Alhaji, Erislene S. Almeida, Camila Rodrigues Carneiro, César Augusto Sodré da Silva, Simone Monteiro, Jane Sélia dos Reis Coimbra

**Affiliations:** 1 Department of Food Technology, Universidade Federal de Viçosa, Avenida PH Rolfs, s/n, Viçosa 36570-000, Minas Gerais, Brazilcesar.sodre@ufv.br (C.A.S.d.S.); 2 Department of Food Science and Technology, Faculty of Agriculture and Agricultural Technology, Aliko Dangote University of Science and Technology, Wudil P.O. Box 3244, Nigeria; 3 Department of Mechanical Engineering, Faculty of Technology, University of Brasilia, Brasília 70910-900, Brazil; 4 Department of Chemistry, Universidade Federal de Viçosa, Avenida PH Rolfs, s/n, Viçosa 36570-000, Minas Gerais, Brazil; camila.r.carneiro@ufv.br; 5 Institute of Chemistry, Federal University of Goiás, Goiânia 74690-900, Goiás, Brazil

**Keywords:** palm tree, oil separation, fatty acids, environmental impact

## Abstract

Palm oil, derived from *Elaeis guineensis*, is a critical component of the global edible oil and industrial fat market. This review provides a comprehensive overview of the sustainability of the palm oil chain, focusing on industrial applications, environmental implications, and economic sustainability. The processing of palm oil, from fruit pulp to refined oil, is detailed, highlighting the importance of refining in maintaining quality and extending application ranges. While palm oil offers health benefits because of its rich fatty acid composition and antioxidant properties, its production poses significant environmental challenges. This review underscores ongoing efforts to balance technological and culinary demands with environmental stewardship and sustainable economic growth. Emerging trends, including interspecific hybrids such as *E*. *guineensis* and *E. oleifera*, are discussed for their potential to increase sustainability and productivity.

## 1. Introduction

**Figure 1:**
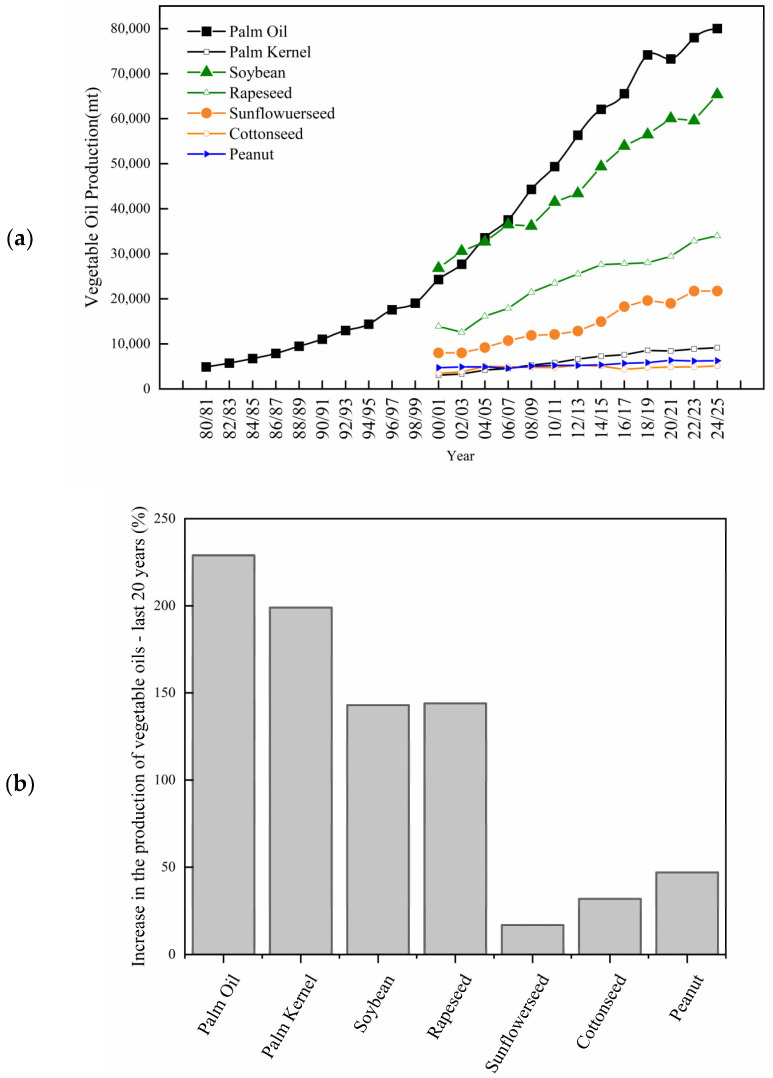
Vegetable oil production worldwide until 2024/2025. (**a**) Evolution of the production of vegetable oil over the last four decades; and (**b**) increase in the production of vegetable oil in the last 20 years [1].

The production of vegetable oils such as coconut, cottonseed, olive, palm kernel, peanut, rapeseed, sunflower, soybean, and palm oils reached a combined total of 223.8 million metric tons (mt) in 2024. Soybean and palm oils were the most common oils produced in recent years [1] (Figure 1).

Considering the production trends from 2000 to 2001 until now, palm oil production increased by 229%, whereas soybean oil production increased by 143% (Figure 1b). In the 2024/2025 biennium, the world production of palm oil is expected to exceed 80 mt. This substantial growth underscores the importance of palm oil, which became a cornerstone of the global edible oil and industrial fat market, now comprising more than 35% of global vegetable oil production [2,3]. Despite occupying only 5.5% of the cultivated land for oils and fats worldwide, palm oil accounts for 32% of the total production [4]. This efficiency positions palm oil as a critical component of the agricultural sector, reflecting its economic importance and versatility. It is used in various products, including shorteners, vanaspati frying fats, margarine, and confectionery fats [5,6].

The increasing consumption of vegetable oils is driven by population growth, the search for renewable energy sources to reduce greenhouse gas emissions, and the expansion of the biofuel sector. These factors collectively establish palm oil as the most widely used vegetable oil worldwide, with high productivity per planted area [7,8]. The yield of oil palm, reaching 4 to 4.5 tons per hectare, far surpasses that of other oilseeds, reinforcing its global dominance [9]. The growing demand for palm oil is also attributed to its diverse applications beyond traditional food use, including its role in biodiesel production, which positioned palm oil as a critical resource for both the food and energy sectors [2].

Owing to its rich fatty acid composition (oleic and palmitic acids), palm oil’s unique semisolid or solid state at room temperature enhances its versatility in various food and nonfood applications. This property enables palm oil to be blended or interesterified with other oils to create trans-fat-free products, which are increasingly important to the food industry [8,10,11]. Furthermore, the refining process of palm oil is critical for maintaining its quality and extending its application range. It removes unwanted compounds while preserving beneficial components such as tocopherols [12,13,14,15].

In this context, the present work aims to provide a comprehensive and up-to-date overview of palm oil processing, focusing on its sustainability. This review synthesizes current knowledge regarding characteristics and processing and highlights emerging trends and challenges in the palm oil industry, including official regulations, environmental issues, and the pursuit of sustainable production practices.

## 2. Oil Palm Tree

The oil palm tree, scientifically known as *Elaeis guineensis*, is often called the African oil palm and belongs to the *Arecaceae* family, sharing its botanical lineage with coconut and date palms [2]. It is a pivotal component of the global palm family, holds significant economic importance, is widely studied, and is commercially exploited [3]. The species *E. oleifera* or *E. melanococca* (also known as “Caiaué”) is known as American oil palm tree. The hypothesis is that the separation of the American and African continents in prehistoric times led to the evolution of those species known today [2]. Researchers generally agree that the oil palm *E. guineensis* is native to Africa’s western and southwestern regions, particularly the area between Angola and Gambia [4]. It is believed that it was domesticated in its native habitat, likely in Nigeria, and spread across tropical Africa over 5000 years ago [5].

**Figure 2:**
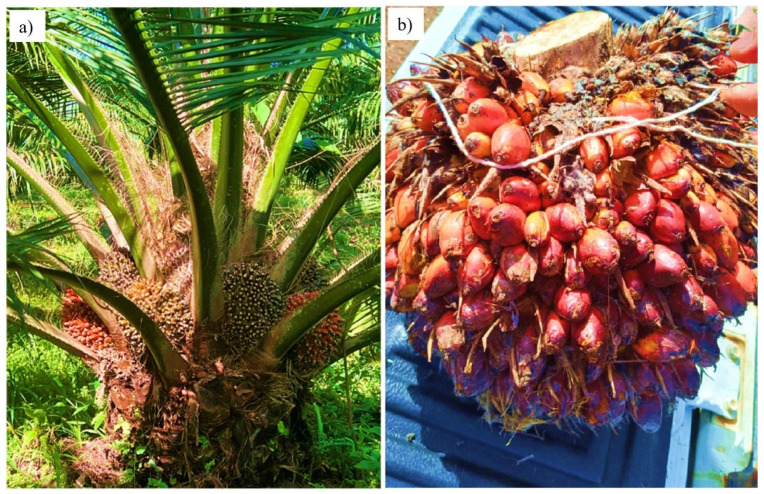
Palm tree (**a**) and fresh fruit bunch (**b**) (source: the authors, 2024).

**Scheme 1:**
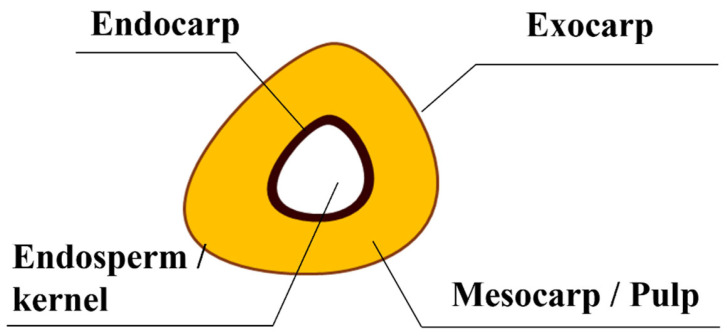
Part of palm fruit.

The oil palm fruit (OPF), originating from the palm tree, is a drupe formed in tight, spiky bunches [6]. Oil palm produces fruits in fresh bunch (FFB) clusters (Figure 2). These bunches are composed of tightly packed spikelets containing fruits and can weigh up to 50 kg, bearing anywhere from a few hundred to a few thousand fruits [16]. Each fruit consists of distinct layers, including an outer skin (exocarp), a fleshy pulp (mesocarp), a protective shell (endocarp), and an inner kernel (endosperm), Scheme 1 [6,7]. The mesocarp, a fibrous matrix, contains palm oil, whereas the kernel harbors oil within its central nut. A typical palm fruit measures approximately 3.5 cm in length and weighs approximately 3.5 to 4.0 g. OPF is recognizable by its reddish color and bunch-like growth pattern. Each fruit consists of two main parts: the oily, fleshy layer, known as the mesocarp, and a single seed, the palm kernel or endosperm. The oil extracted from the mesocarp is referred to as crude palm oil (CPO), whereas the oil from the kernel is called palm kernel oil [6].

**Figure 3:**
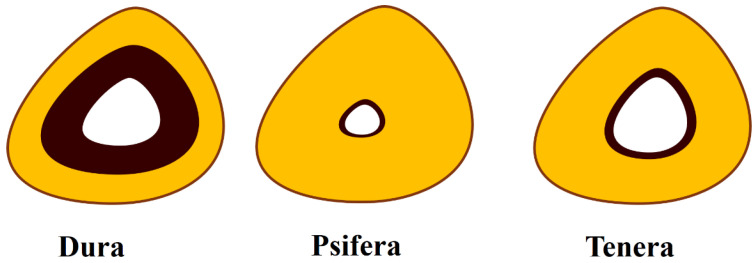
Classification of oil palm according to endocarp thickness. Adapted from [9].

The African oil palm can be classified into dura, pisifera, and tenera according to the thickness of the endocarp covering the kernel (Figure 3). Dura is the predominant type, with a frequency of approximately 97% in wild palm groves; tenera is a hybrid of dura and psifera [2,8,9].

The most commercially important oil palm species is the African palm (*Elaeis guineensis*), which is cultivated worldwide, mainly in Indonesia and Malaysia [17]. The most significant exploitation of this species is its high oil productivity per planted area. The American oil palm (*Elaeis oleifera*) is distributed across various regions of Central and South America. Unlike the African oil palm, it is not commercially exploited due to its lower productivity and is utilized by traditional communities for domestic consumption [12]. However, this species has several advantages over African palm: greater resistance to diseases such as *fusarium* wilt and pests such as fatal yellowing [10,18] and lower trunk growth in height, making it easier to handle and harvest bunches [11]. This American species also has a high carotenoid content (over 4600 ppm), which is higher than that of African species (between 600 and 1000 ppm) [12,18]. Only the shell dura type exists in the *E. oleifera* species [13,14]

The interspecific hybrid *E. guineensis* × *E. oleifera* is currently gaining prominence. This species produces hybrid or high oleic acid palm oil (HOPO) [15,19]. This cultivar combines the advantages of its two parent species. These include lower vertical growth, greater resistance to diseases and pests, high productivity per planted area, and a distinctive composition rich in unsaturated fatty acids and antioxidant compounds such as carotenes. Furthermore, hybrids exhibit lower acidity levels due to reduced lipase activity, which are enzymes that become active when fruits are harvested improperly [20].

## 3. Features of Palm Oil and Its Bioactivity

A striking characteristic of palm oil is its oleic acid content and concentration of antioxidant compounds, such as carotenes and tocols, making it more resistant to oxidation and more suitable for frying, meeting the requirements of the food industry [13]. African palm oil (APO) is extracted from the fruit’s mesocarp and is known for its unique composition of fatty acids. It presents a balanced profile of saturated and unsaturated fatty acids, which makes this oil extremely versatile and enables a wide range of applications [21,22]. APO contains approximately 44% palmitic acid, 40% oleic acid, 10% linoleic acid, and 5% stearic acid [12,13]; thus, it contains approximately 50% saturated fatty acids and 50% unsaturated fatty acids [21]. This composition differs from that of the oil obtained from the American species *Elaeis oleifera* (Caiaué). Among *E. oleifera* palm trees, the content of unsaturated fatty acids varies from 47% to 69% for oleic acid, 2% to 19% for linoleic acid, 0.1% to 1.2% for linolenic acid [23], and the content of palmitic acid is approximately 24% [24]. The interspecific hybrid has a fatty acid profile composed of oleic acid (55%), palmitic acid (27%), and linoleic acid (11%) [12].

Palm oil is often criticized for its high concentration of saturated fatty acids (SFA), particularly palmitic acid, which are linked to health issues such as obesity, cardiovascular diseases, diabetes, and cancers. However, recent work showed that palmitic acid of plant origin has a negligible effect on increasing total blood cholesterol and low-density lipoprotein cholesterol levels compared with palmitic acid of animal origin and that palm oil does not induce increases in biomarkers related to the risk of cardiovascular diseases in relation to unsaturated fatty acids and, in general, does not increase the risk of obesity, diabetes, cancer, or obesity [25,26,27,28]. Another fatty acid present at high concentrations in palm oil is oleic acid, a non-unsaturated fatty acid (MUFA) that can reduce harmful cholesterol levels and protect against heart disease [12,20,21] and poly-unsaturated fatty acid (PUFA). These unsaturated fatty acids are also called omega (ω), according to the position of the carbon where the unsaturated fatty acid is located, which can be ω-3 (linolenic acid), ω-6 (linoleic acid), and ω-9 (oleic acid) fatty acid [29]. Significantly, moderate palm oil consumption may not be associated with an increased risk of developing these health issues.

Palm oil is mainly composed of a mixture of triacylglycerols, approximately 95% [29]. Nevertheless, the oil contains various minority components, including free fatty acids (FFA), monoacylglycerols (MAG), diacylglycerols (DAG), metals, phospholipids, peroxides, and chlorophylls, as well as antioxidants and high-value compounds such as carotenoids, vitamin A precursors, tocols (tocopherols and tocotrienols), and phenolic compounds [30,31,32,33]. Vegetable oils are essential in the human diet because they are important carriers of fat-soluble vitamins such as A, D, E, and K [34].

Carotenoids, which are among the most essential minor components of palm oil, feature long chains with conjugated double bonds that significantly influence the color of the oil, ranging from yellow to orange-red [35]. They are liposoluble pigments responsible for the distinct orange color of the oil extracted from the mesocarp. The concentration of these pigments in the oil obtained from fruits of *E. guineensis* varies between 600 and 1000 ppm, whereas for *E. oleifera*, it is above 4000 ppm. In the case of interspecific hybrids, the concentration ranges from approximately 1400 to 2300 ppm [19,21,36]. Approximately 90% of the carotenes present in the oil are α- and β-carotenes [19,36].

Carotenoids have antioxidant properties that positively affect human health, making palm oil valuable for preventing vision problems, cardiovascular disease, and cancer [37,38,39,40]. Carotenoids also serve as precursors to vitamin A, with β-carotene exhibiting the greatest provitamin A (retinol) activity. In addition to their beneficial effects on health, carotenes have a significant effect on the oxidative process of the oil, as they can reduce oil oxidation due to their potential to suppress ^1^O_2_ (singlet molecular oxygen), and this ability increases according to the number of double bonds in the chain [34,41]. In addition to their nutritional value, these compounds are removed from the oil during the refining process to obtain an oil with a lighter color for greater consumer acceptance in various industrial purposes [42]. On the other hand, maintaining residual carotenoids in postbleaching palm oil is essential, as they slow the oxidation process [41].

Another essential minor component in palm oil is tocopherols and tocotrienols (collectively known as tocols). Together with carotenes, these compounds can act synergistically as antioxidants, enhancing the oxidative stability of the oil [17]. These compounds have a chromanol group that affects vitamin E activity in the diet [43]. A lack of this vitamin can cause anemia, a decreased immune response, retinopathy, neuromuscular and neurological problems [44], and potent anticarcinogenic substances and help combat thrombosis [45]. In addition to carotenes and tocols, palm oil is rich in compounds with important biological activities [45] that improve the absorption of nutrients and support brain function, including phospholipids, phenolic compounds and significant amounts of squalene and phytosterols [46,47,48]. Table 1 shows some studies that address these nutritional components of the oil. Saturated fatty acids in palm oil are often criticized for their potential health impacts, such as obesity and cardiovascular diseases. However, recent studies suggest that plant-derived palmitic acid has a negligible effect on increasing total blood cholesterol and low-density lipoprotein cholesterol levels compared with animal-derived palmitic acid [25,26,27,28]. Unsaturated fatty acids, particularly oleic acid, are known for their health benefits, such as reducing harmful cholesterol levels and protecting against heart disease [12,13,14,15,19,20,21,22,23].

**Table 1:** Health benefits of bioactive compounds found in oil palm.

Bioactive Compound	Content Range	Health Benefits	Reference
Carotenoids	600–1000 ppm (*E. guineensis*) 4000+ ppm (*E. oleifera*)	Provitamin A activity	[49,50,51,52]
		Protection against cardiovascular disease	[38,51,53,54]
		Anticancer activity	[51,55,56,57,58,59]
		Antioxidant	[40,51,55]
Phytosterols	100–200 ppm	Phytosterols help reduce LDL cholesterol levels	[60,61]
		Anticancer characteristics	[62]
Tocotrienols and Tocopherols	600–1000 ppm	Vitamin E activity	[57,63,64,65]
		Reduces the risk of high cholesterol, risk of developing cancer, cardiovascular diseases, brain disorders and increases immunity	[57,63,65,66,67]
		Antioxidant	[44,64,65,68,69,70]
Phenolic acids	10–50 ppm	Anti-inflammatory	[71,72]
Phospholipids	10–50 ppm	Improves nutrient absorption and digestion	[63,73]
		Energy endurance	[74,75]
		Brain development	[76,77]
Squalene	200–500 ppm	Anticancer activity	[78]
		Protection against cardiovascular disease	[79]
		Delay in the production of cholesterol	[80,81]
Saturated fatty acids	Palmitic acid: Approximately 44% in *E. guineensis* and 24% in E. oleifera Stearic acid: Approximately 5% in *E. guineensis* [16,17,18,19,20,21,22,23,24,25]	Palmitic: essential fatty acids in cell membrane, transportation, secretary lipids and part of the human body and energy production	[25]
		Vegetable palmitic acid has a negligible effect on blood cholesterol levels	[12,13,14,15,19,20,21,22,23,24,25]
Unsaturated fatty acids	Oleic acid: Approximately 40% in *E. guineensis*, 47–69% in *E. oleifera*, and 55% in interspecific hybrids Linoleic acid: Approximately 10% in *E. guineensis*, 2–19% in *E. oleifera*, and 11% in interspecific hybrids	Modulates physiological functions, inhibits cancer proliferation, reduces inflammation, reduces blood pressure and improves wound healing	[6,82]
		Beneficial effects on anti-inflammatory and autoimmune diseases	[6,12,13,14,15,19,20,21,22,23]

## 4. Processing of Crude Palm Oil

After the third year of planting, the first bunches of fruits begin to ripen. Approximately 180 days after the start of inflorescence development, the oil begins to form, with its formation accelerating notably after two weeks of maturation [83]. Two types of oil are extracted from the fruit of the palm tree: red crude palm oil from the mesocarp and palm yellow crude kernel oil from the endosperm, each of which has a distinct composition [84]. Mesocarp oil is primarily used for edible purposes, whereas palm kernel oil has applications in the oleochemical industry [85]. When fully ripe, the fruit mesocarp typically contains 68.0% to 73.2% (*w*/*w*) edible oil [85].

**Figure 4:**
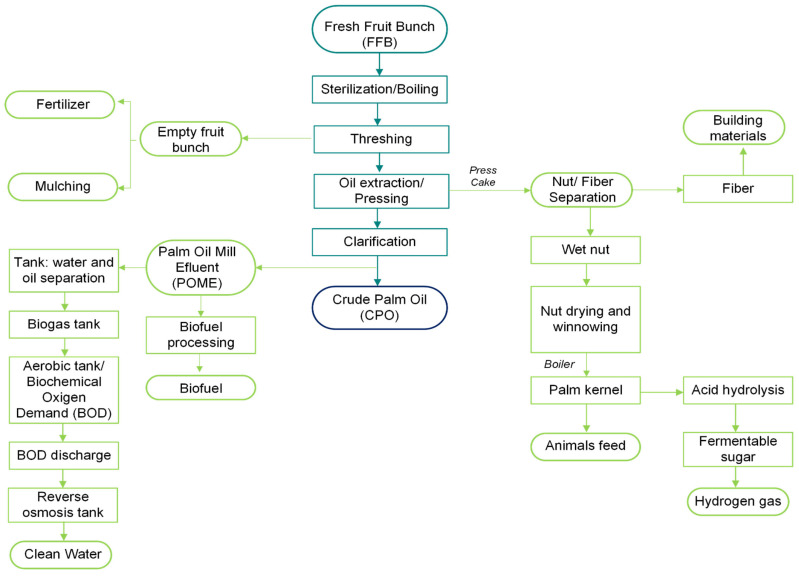
Flowchart of crude palm oil extraction from fresh fruit bunches (light blue), extraction residues and their bioproducts (green), and the final product (dark blue). Adapted from [100,105].

The production of CPO involves various complex steps [9], which include sterilization of fresh fruit, fruit detachment, digestion, oil extraction, and clarification [83] (Figure 4). Sterilizing fresh fruit is a critical step involving moisture absorption and heat treatment to deactivate lipolytic enzymes such as lipases in the fruit mesocarp [86,87,88]. These enzymes can otherwise lead to increased levels of free fatty acids (FFAs) [83], causing quality issues during storage, processing, and refining [89]. The condensed water from this process is a significant source of palm oil mill effluent (POME). Several studies focused on optimizing the use of POME, such as reusing the water generated during oil extraction in milling processes or as drinking water [90]. Additionally, POME is used for biogas generation [90].

Palm oil production can be categorized into artisanal and industrial milling methods. The oil extraction method, which employs various techniques, is crucial in determining the yield and quality of oil. These extraction methods can be classified based on their complexity and processing capacity, ranging from artisanal techniques and small mechanical units to medium-scale and large industrial mills [91].

Artisanal palm oil extraction represents the oldest method of oil separation and is often conducted with traditional equipment. In artisanal extraction, harvested fruit bunches are left for several days to facilitate the detachment of the fruits before the oil extraction process, increasing lipase activity and leading to the hydrolysis of palm oil triglycerides [92]. The fruits are subsequently boiled in a drum, and extraction is performed via a manual or motorized press.

Industrial palm oil extraction employs two primary methods: chemical or wet techniques, such as solvent extraction, and physical or dry methods, such as mechanical pressing. These methods can achieve oil extraction efficiencies ranging from 75% to 90% [93,94,95]. The choice between these two methods depends on several factors, such as the quality and acidity of the crude oil or local legislation [96].

During the solvent extraction process, oil is extracted from the ruptured cells of the oil palm via water or steam. This process coagulates proteins and hydrolyzes any starch, glue, or gum that may be present [97]. These substances can cause oil foam during frying. The alkaline neutralization stage of chemical refining removes free fatty acids and most phosphatides. In the subsequent oil clarification step, hydrolyzed and coagulated products are removed. After moisture evaporation, extracted crude palm oil (CPO) is obtained [98,99].

Dry extraction, on the other hand, uses a hydraulic press, screw press, or centrifugation to break the oil cells. The screw press is typically more suitable for continuous extraction systems, whereas the hydraulic press is commonly used in batch or semi-batch extraction systems. After being pressed, the crude palm oil is separated from the fibrous mesocarp, with the remaining fiber components retaining approximately 5 to 6% (*w*/*w*) of the oil. The yield and quality of the extracted oil are influenced by factors such as the initial oil and moisture contents, operating temperature, heating time, and applied pressure [99]. The pressure is typically reduced to prevent fruit kernel breakage, which increases oil retention to around 10–12% in the mesocarp biomass [100].

High-grade palm oil typically has low free fatty acid and moisture levels, minimal contaminants, and excellent deterioration of the bleachability index (DOBI). The grade and market value of palm oil depend on the quality of the extracted product. Triacylglycerol (neutral lipid), carotenoids, phytosterols, and vitamin E (tocopherol and tocotrienols) are desirable components of oils because of their nutritional value. However, during extraction processes, whether artisanal or industrial, various compounds are extracted alongside the oil, including FFA, partial acylglycerols, phosphatides, sterols, tocopherols, tocotrienols, hydrocarbons, pigments, vitamins, sterol glycosides, protein fragments, traces of pesticides, dioxins, and heavy metals [49].

Consequently, CPO contains undesirable compounds such as water, oil impurities, and fruit fragments. Reducing these compounds is crucial to ensure the quality of palm oil and expand its range of applications. The objectives of the refining process include achieving a moisture content below 10% and reducing the FFA level to 0.3% [101]. Conversely, free fatty acids, phospholipids, and gums are considered contaminants and are undesirable from a chemical standpoint [102].

**Figure 5:**
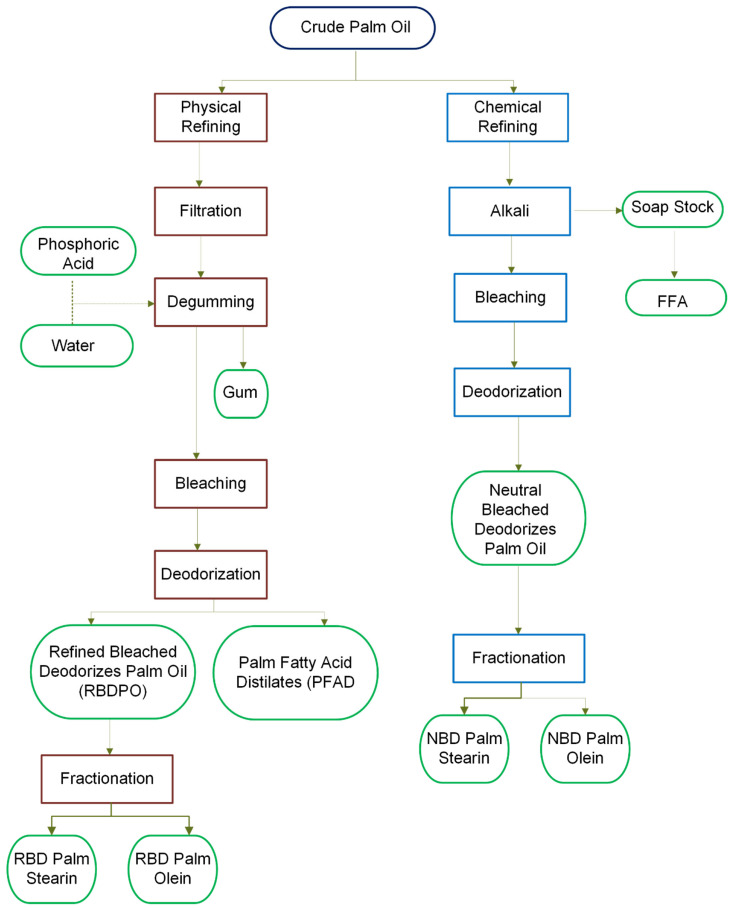
Refining methods for CPO: chemical (brown) vs. physical methods (blue) and final products (green). Adapted from [106].

CPO must undergo refining to have the desired purity characteristics and become edible [30]. During the refining process of CPO, which may be chemical or physical, these impurities are effectively removed, resulting in refined, bleached, and deodorized (RBD) palm oil [49], as depicted in Figure 5. The quality of refined palm oil is primarily assessed based on criteria such as the free fatty acid content, iodine value, peroxide value, moisture content, saponification value (SV), and impurity level.

Chemical refining involves removing free fatty acids by alkali and separating the soap by centrifugation (sludge). When chemically refined, CPO is washed with a sodium hydroxide or sodium carbonate solution to reduce free fatty acids and remove phospholipids and other polar lipids [103]. However, alkali refining alone may not eliminate all potentially undesirable chemical components [104].

Physical refining removes free fatty acids and other compounds through a stripping process. The choice of refining method depends on the characteristics of each oil. Oils such as palm, palm kernel, and coconut, which have low levels of phospholipids, are almost always physically refined [107].

Physical refining has advantages in its use of fewer chemicals and the production of fewer effluents. For crude palm and palm kernel oils with low initial phosphatide contents and high carotene and free fatty acid (FFA) contents, physical refining is preferred because it reduces the loss of neutral oil and operational costs [30,45,49]. Processing parameters can be adapted to retain better desirable secondary components such as tocopherols and tocotrienols and minimize the production of unwanted trans fatty acids [44].

When physically refined, CPO undergoes bleaching and deodorization, which require high temperatures [41]. Bleaching is an adsorptive process conducted from 95 °C to 135 °C [49]. During this process, neutral or acid-activated bleaching earth removes pigments, metals, oxidation products, and soaps [17]. Some carotenes are removed during bleaching and the remaining carotenes are destroyed during deodorization at temperatures of 240 °C or higher [21]. This heat bleaching step removes free fatty acids, aldehydes, and ketones through volatilization. Oils with low DOBI and high levels of FFA and peroxides are more prone to bleach [108]. The DOBI value is the ratio of oil absorption at 446 nm to 268 nm, indicating the relative amounts of carotenes and oxidized carotenes. A higher DOBI value signifies fewer oxidized carotenes, making the oil easier to bleach. DOBI values between 2.5 and 4.0 indicate average to good crude oil quality, whereas values below 2.0 indicate poor quality, which is difficult to bleach [48].

The high temperatures used in refining processes may lead to undesirable chemical changes due to elevated temperatures in the refining steps [42]. The use of bleaching earth can lead to the formation of oxidation products, increasing the levels of FFA’s, foams, color and viscous compounds [40,42]. Other unwanted products formed during refining are 3-monochloropropane-1,2-diol (3-MCPD) and glycidyl esters (gEs), which are process-induced contaminants [108,109]. These compounds are toxic, and their consumption is linked to the formation of tumors.

The formation of 3-MCPD and gEs is influenced primarily by temperature, especially during oil deodorization [108,110]. 3-MCPDs are virtually undetectable in virgin, unrefined oils [111]. These contaminants are formed not only in oils during processing, but also in ready-to-eat foods such as bread, cakes, cookies, cereals, roasted coffees, and baby foods [109,110,112]. In addition to the conditions mentioned above, several other factors influence the formation of these compounds, such as the type of soil, fertilizers, and harvest interval of the bunches [111]. The formation mechanisms of these contaminants are not yet completely elucidated [111,113], but ways to mitigate their formation are known: reducing chlorides and other precursors by washing the crude oil before the deodorization stage [114,115]; controlling the DAG content and reducing the exposure and time to high temperatures during processing [111,116]; and using neutral bleaching earths since acid-activated earths undergo treatment with hydrochloric acid [116].

During transport, storage, and consumption, refined oil may gradually change color, becoming darker shades [117], in a phenomenon called color reversal. Color reversal is generally associated with poor oil quality or inadequate degumming and bleaching processes [40,44,48]. The presence of colored pigments and oxidation compounds has an important effect on the final color of the oil and plays a role in this phenomenon of color reversal. If, on the one hand, temperature leads to the discoloration of carotenes, it also favors oxidation, which leads to the formation of other types of colored compounds. There may be an increase in the yellowish or red color of the oil because the formation of tocopherol oxidation products such as γ-tocopherol and γ-tocopherol-5,6-quinone may also lead to the stabilization of other pigments against their removal by adsorption [48,117,118].

## 5. Utilization of Palm Oil

Palm oil can be used for a variety of purposes. There are several uses for mesocarp oil and kernel seed oil; approximately 80% are for food purposes, and the remaining 20% are used as feedstocks for various nonfood applications [119,120]. At the household level, palm oil has been used for domestic cooking in Southeast Asia, tropical Africa, and South America for centuries [9,121]. The food industry adopted palm oil in its refined form in recent decades because of its functional benefits, versatility, and widespread availability. The main advantages of palm oil include (1) its high stability over time because palm oil helps maintain the product’s taste throughout its shelf life because of its higher oxidation stability than other vegetable oils do [122]; (2) its neutral taste and the smell of deodorized palm oil, allowing it to be incorporated into a variety of foods without affecting flavor; this neutrality ensures that the oil does not mask the flavors of other ingredients, such as milk, cocoa, and hazelnuts [123]; (3) versatility as a vegetable fat due to the possibility of fractionation into different solid contents, making it suitable for different requirements of texture and flavor in the final products [124]; (4) smooth and creamy texture since food products with palm oil have an excellent mouth feel with specific characteristics for each product; for example, palm oil contributes to chocolate spreads’ smooth and creamy texture and spreadability [123]; and (5) as an alternative to trans-fat, palm oil is a suitable replacement for partially hydrogenated fat [124]. A high percentage of the products sold at supermarkets use palm oil in their formulation. These products include margarine, confectionery, ready-to-eat meals, food snacks, chocolate, ice cream, bakery products, and nonfood products such as soap, candles, and cosmetics [125].

The fractionation process can determine the chemical and physical properties of olein and stearin: at the industrial level, refined, bleached, and deodorized (RBD) olein is mainly used in food products such as cooking and frying oils, shortening, and margarine; the RBD stearin is also used to make margarine and shortening [12]. Unfractionated RBD palm oil makes ice cream, margarine, shortening, vanaspati (vegetable ghee), frying fats, and ice cream [123].

Nonfood uses of palm oil include cosmetics and personal care, soap, candles, pharmaceuticals, metal plating, lubrication and grease, surfactants, industrial chemicals, agrochemicals, coatings, paints, lacquers, electronics, leather, and biodiesel production [100].

In addition to mesocarp and kernel oils, which are the main oil palm products, tree and fruit processing waste have several uses. Sludge is used in traditional soaps and fertilizer, and palm kernel cake is widely used as an input into the feed industry and fertilizer. The processing wastes, namely, empty bunch refuse, fibers, shells, sludge, and mill effluent, constitute approximately 75% of the total mass of the oil products. The other parts of the palm tree (trunk, leaves, and fiber) have broad uses, while the bunch refuse and byproducts from oil processing (fiber, shell, and sludge) can be used as fuel for mills, making briquettes a substitute for fuel wood. Kernel cake was applied in animal feed and organic fertilizer production as a substrate for mushroom production. The midribs and rachises are applied as roofing materials [121].

## 6. Impact and Sustainability of the Palm Oil Chain

Since the beginning of the 21st century, approximately 5 million hectares of forests were deforested annually. Brazil and Indonesia are critical areas for deforestation, accounting for 33% and 19% of deforested areas, respectively [126]. The land used for oilseed cultivation increased from 170 MHa in 1961 to 425 MHa in 2017 [127]. This increase is due to the current demand for renewable energy sources and the needs of both the food and nonfood industries. Palm cultivation stands out in this increase in production owing to its lower price and higher productivity than those of other vegetable oils [128].

The relationship between oil palm cultivation and the environment is quite controversial because of the social and environmental impacts versus the opportunities generated by this culture [127]. The most common environmental impacts are deforestation; a reduction in woody biomass; the drainage of peatlands; and impacts on biodiversity, water quality, and increased greenhouse gas emissions and haze when fires are used [126,127,128]. This culture also requires a large amount of labor to address field activities such as planting, cultivating, harvesting, collecting and processing bunches [128], which leads to social problems such as labor exploitation, low wages, social inequality and compromised well-being at the village level; another social problem is land grabbing and conflicts [127,129].

Approximately 50% of products present in supermarkets have palm oil in their composition, in addition to its direct use in the production of food, feed, fuel, cosmetics, detergents, and the chemical industry [130]. Therefore, despite these negative aspects, boycotting palm oil cultivation is not viable, especially considering its significant industrial, economic, and social importance and yield per planted area. Among the two largest vegetable oil crops, palm yields 2.93 MT/HA, whereas soybean yields only 0.46 MT/HA. The superiority in terms of palm yield becomes more evident when we consider that, to achieve this yield, oil palm was planted at 27.41 (1000 HA) and soybeans at 143.35 [131].

Instead of boycotting, promoting sustainable oil palm cultivation is the path to pursue. Initial steps were taken; according to Basiron and Weng [132], much time was spent understanding and managing the palm oil industry’s economic, environmental, and social aspects, with sustainability aspects only recently being integrated into business strategies. Currently, it is desired to produce certified palm oil to integrate production and sustainability. These certificates are procedures by which guarantees are provided that a product, process, or service along the supply chain complies with certain standards [132]. In this context, the Round Table on Sustainable Palm Oil (RSPO) plays a crucial role. RSPO round tables are private agreements aimed at enhancing the sustainability of the global palm oil supply chain [130].

RSPO is a global nonprofit organization with volunteer members aiming to transform the palm oil industry into a sustainable industry. To this end, stakeholders should be integrated across the entire palm oil production chain to develop and implement global standards for sustainable palm oil. According to the organization’s data from 2023, there were nearly 5000 hectares of certified areas, more than 4000 companies, and approximately 7000 facilities with supply chain certificates. Additionally, this year, more than 14,000 tons of sustainable palm oil were produced [129].

RSPO is the most recognized and accepted international sustainability certification today. However, there are national certifications, such as the Malaysian Sustainable Palm Oil (MSPO) and Indonesian Sustainable Palm Oil Standard (ISPO), which are certifications from Malaysia and Indonesia, countries that together contribute approximately 90% of global palm oil production [133]. These certifications ensure that palm oil plantations in Malaysia and Indonesia are managed following good agricultural practices [134]. Table 2 presents several certification schemes and legislation that aim to sustain palm cultivation.

Sustainable production protects the natural environment while improving business operations and sharing economic growth with the local community through employment and fair trade [135,136]. The sustainability of palm oil can be assessed by considering three main aspects: economic, ecological and social sustainability [133,137].

There are several economic benefits, such as agricultural development, increased investment and employment in rural industry, and international competitiveness, which lead to positive financial and socioeconomic impacts on the immediate surroundings of plantations. On the other hand, the economic development of producing countries resulted in social and environmental losses [135]. One way to mitigate these losses would be to invest in the education and training of small producers since it is estimated that they are responsible for cultivating approximately 50% of the global palm oil area [135,138]. The integration of these producers into certified palm oil cultivation is essential, as it results in increased agricultural income and employment and reduced poverty rates at the local, regional, and national levels [135].

Environmental factors are the most critical of the three pillars since developing palm oil cultivation requires many natural resources, which impact the environment through greenhouse gas emissions, deforestation, and loss of biodiversity [136,137]. However, the contribution of the palm oil sector to driving deforestation remains inconclusive [137,139]. In any case, to continue meeting the current needs of the food and nonfood industries, sustainability standards were established by local authorities and NGOs to ensure transparency and control over the operations of the palm oil supply chain, from palm oil plantations to obtaining final products [133].

**Table 2:** Principles and objectives of the main national and international legislation and certificates related to sustainable oil palm cultivation.

Coverage	Certifications/ Legislation	Key Aspects	Ref.
International Certifications	RSPO—Round Table on Sustainable Palm Oil	Behave ethically and transparently Operate legally and respect rights Optimize productivity, efficiency, positive impacts and resilience Respect Community and human rights and deliver benefits Support smallholder inclusion Respect workers’ rights and conditions Protect, conserve and enhance ecosystems and the environment	[133,134]
	ISCC—International Sustainability and Carbon Certification	Certification of palm oil used as a feedstock for biofuels Preservation of natural areas characterized by their high biodiversity or that can store carbon (High Conservation Value = HCV) Application of good agricultural practices, for example, to maintain soil fertility or preserve water quality and to reduce the use of pesticides Safe working conditions are maintained, for example, through employee training and the provision of appropriate protective clothing Compliance with labor and human rights laws and ensuring responsible working conditions that promote health Compliance with applicable laws and regulations Adherence to good management practices	[136,137,139]
	POIG—Palm Oil Innovation Group	Creates and promotes innovations in the palm oil industry Support the RSPO principle Seeks the adoption of responsible palm oil production practices by key supply chain participants through the development and sharing of a reliable and verifiable benchmark	[140]
	RSB—Roundtable on Sustainable Biomaterials; SAN—Sustainable Agriculture Networks	Ensure the inevitable transformation to a biocircular economy is environmentally sustainable and socially fair. Transforming agriculture for the greater good of all Positive change across agricultural value chains and working to create lasting impact	[136,137,141,142]
Malaysia	MSPO—Malaysia Sustainable Palm oil	Management commitment and responsibility Transparency Compliance with legal requirements Social responsibility, safety and employment conditions Environment, natural resources, biodiversity and ecosystem services Best practice Development of new plantings	[133,134]
	MPOB—Malaysia Palm Oil Board	To enhance and support the well-being of the Malaysian oil palm industry through the dissemination of timely, reliable and comprehensive data and economic research findings as well as market information To conduct research on the economics of production, downstream processing, and marketing of the Malaysian palm oil industry. To ensure compliance with conditions imposed on the license regarding the registration of contracts and submission of monthly statements (PL forms). To disseminate comprehensive, accurate and timely industry and market information. To provide inputs for the establishment of the national palm oil development policy.	[143]
Indonesia	ISPO—Indonesia Sustainable Palm Oil	Plantation Management Protection of the utilization of Primary Forest and Peatlands Environmental Management Responsibility for Workers Responsibility for Social and Economic Empowerment Continuous Business Improvement	[133,134]
Brazil	Federal Law No. 12,651/2012	Establishes general rules on the Protection of Native Vegetation, including areas where oil palm can be cultivated; forest exploitation; the supply of forest raw materials; the control of the origin of forest products; the control and prevention of forest fires, and the provision of economic and financial instruments to achieve its objectives.	[144]
	Decree n°7172/2010	Agroecological zoning of oil palm cultivation in Brazil to be applied from the 2010/2011 harvest Guide the expansion of Brazilian palm production on a technical-scientific basis, to guarantee sustainability in its economic, social and environmental aspects. Offer sustainable economic alternatives to rural producers in the region and provide a basis for planning sustainable land use following current legislation. Promote land use planning in the region’s anthropized areas following each state’s Ecological and Economic Zoning. Provide a basis for planning development hubs in rural areas that align with the public policies of the different levels of government.	[145]
European Union	Regulation (EU) 2018/841	Relates to reducing greenhouse gas emissions from land use activities and achieving the long-term climate targets of the Paris Agreement Deals with the European Union’s strategy to combat deforestation and promote the sustainable use of agricultural products Establishes requirements to ensure that palm oil used in the EU does not contribute to deforestation and forest degradation	[146]
United States	Lacey Act	Deals with the Importation of Products: Prohibits all trade in plants and plant products from illegal sources in any state of the United States and other countries. Requires importers to declare the country of origin and species name of all plants contained in their products	[147]

It is possible to associate a risk factor in the long term with investing in industries that prioritize economic factors and precarious environmental and social factors [148]. Therefore, as ways of combining the growth of palm cultivation with social development, small producers are assured of secure land titles, access to credit and technical support, and decent and fair working conditions, and given the importance of small farmers [136,149].

In addition to production considering these three pillars of sustainability, other mechanisms, such as improving productivity by applying better cultivation practices and quality inputs; planting conditions; fertilizer application; harvesting; transportation of freshly collected fruit bunches and loose fruit; weed control; and sanitary control, are also important potential sources of high production costs. These processes can be optimized within the ecologically sustainable development framework, increasing competitiveness [136,150]. In any case, it is important to emphasize that these pillars are closely linked, and it is not acceptable to prioritize one over the others. All actions to improve palm oil cultivation must consider economic development, social development, and environmentally friendly actions.

One way to make oil palm cultivation more sustainable is to grow it in areas that were already deforested, avoiding forest degradation. It is also possible to mitigate the impacts of waste generated, transforming it into coproducts, especially renewable energy resources. The empty bunches can be used for vegetable cover or burned as fuel for boilers, POME can be used as fertilizer, and the trunks and leaves can be chipped and left between the lines as vegetable cover to prevent fires [132]. In factories, fiber, bark, and EFB are burned as fuel for boilers. Residues from the burning of palm bark and fibers in a furnace form palm oil clinker (POC), a hard and porous material that can be added to concrete for the construction of masonry blocks, resulting in a material with the ability to control noise [150]. Owing to continuous research and development into new uses, most waste is now accounted for [132].

Furthermore, positive economic and social impacts can also be highlighted. This culture contributes to economic development and improves well-being, in addition to being a source of employment, which leads to improved living standards, poverty reduction and better income distribution, workers’ accessibility to medical benefits, school facilities for workers’ children, and the development of rural areas [127,128].

The great challenge of oilseed cultivation, especially oil palm cultivation, is to sustainably meet the global demand for this raw material while mitigating its environmental and social impacts.

## 7. Conclusions

This review highlights the critical aspects of palm oil sustainability, processing, and utilization. Palm oil processing involves several key steps, including harvesting, sterilization, threshing, pressing, and refining. These processes are crucial for maintaining the quality and extending the application range of palm oil. The refining process removes unwanted compounds while preserving beneficial components such as tocopherols. The versatility of palm oil is evident in its diverse applications, including food products such as shorteners, vanaspati frying fats, margarine, and confectionery fats, as well as its role in biodiesel production. The ongoing efforts to balance technological demands with environmental stewardship and sustainable economic growth are underscored, emphasizing the importance of sustainable production practices and the potential of interspecific hybrids to increase productivity and sustainability.

## Data Availability

No new data were created or analyzed in this study. Data sharing is not applicable to this article.
